# Determination of Polyphenols, Capsaicinoids, and Vitamin C in New Hybrids of Chili Peppers

**DOI:** 10.1155/2015/102125

**Published:** 2015-10-01

**Authors:** Zsuzsa Nagy, Hussein Daood, Zsuzsanna Ambrózy, Lajos Helyes

**Affiliations:** ^1^Institute of Horticulture, Faculty of Agriculture and Environmental Sciences, Szent István University, Páter Károly Street 1, Gödöllő 2100, Hungary; ^2^Regional Knowledge Centre, Szent István University, Páter Károly Street 1, Gödöllő 2100, Hungary

## Abstract

Six hybrids were subjected to chromatographic analyses by HPLC for the determination of phytochemicals such as capsaicinoid, polyphenol, and vitamin C. The dynamics of ripening of 4 of the hybrids were also characterised. Seven capsaicinoids could be separated and determined; the major compounds were nordihydrocapsaicin, capsaicin, and dihydrocapsaicin, while homocapsaicin and homodihydrocapsaicin derivatives were detected as minor constituents. Capsaicin content ranged between 95.5 ± 4.15 and 1610.2 ± 91.46 *μ*g/g FW, and the highest value was found in Bandai (*C. frutescens*) at the green ripening stage. The major capsaicinoids had a decreasing tendency in Bandai and Chili 3735 hybrids, while no change was observed in Beibeihong and Lolo during ripening. Nine polyphenol compounds were detected including 8 flavonoids and a nonflavonoid compound in the pods of all hybrids. The major components were naringenin-diglucoside, catechin, and vanillic acid-derivative and luteolin-glucoside. Naringenin-diglucoside ranged from 93.5 ± 4.26 to 368.8 ± 30.77 *μ*g/g FW. Except vanillic acid-derivative, dominant polyphenols increased or remained unchanged during ripening. As for vitamin C, its content tended to increase with the advance in ripening in all hybrids included in this study. The highest value of 3689.4 ± 39.50 *μ*g/g FW was recorded in Fire Flame hybrid.

## 1. Introduction

The components evolving pungency in chili peppers have been established as a mixture of acid amides of vanillylamine and C8 to C13 fatty acids, also known as capsaicinoids [1]. Capsaicinoids are secondary metabolites and are synthesised by glands at the join of the placenta and the pod wall of pungent peppers [2]. The effect of capsaicinoids on human health has been widely investigated. For instance, it is beneficial in low concentration against gastric injuries [3], stimulates cation channels (Na^+^, K^+^, and Ca^2+^) in sensory receptor membrane [4], evokes pain, and activates autonomic reflexes [5]. Environmental factors and the circumstance of cultivation influence capsaicinoid content of the pods [6, 7], while probably a higher impact on pungency by the genotype is present [8, 9]. Besides, the amount and proportion of capsaicinoids are changing during the ripening process of the pods [10–13]. Flavonoids represent a significant subgroup of polyphenols [14] and naturally occur in high concentration in wild mint [15] and grape [16] while generally pungent peppers have moderate level of polyphenol content. The health protective attributions of them are mainly associated with preventing cancer through inhibiting certain enzymes and suppressing angiogenesis [17]. The polyphenol content in pungent peppers is found to be influenced by genotype and the ripening process [18–20]. Ascorbic acid, the main component of vitamin C, is very abundant in fresh* Capsicum* species and has been found to be beneficial in maintaining collagen synthesis and healthy immune-system and also has antitumor properties [21–23]. The content of ascorbic acid is highly varying among cultivars and ripening stages [24, 25]; in addition, the utilised agricultural techniques play significant role in the final amount of ascorbic acid in the pods [26].

Numerous cultivars of pungent pepper are nowadays available; however, many of them have not been analysed for their quality and nutritional components. The objective of the present work is to investigate capsaicinoid, polyphenol, and vitamin C content in six hybrids of chili pepper (Bandai, Beibeihong, Lolo, Chili 3735, Fire Flame, and Star Flame) using recently developed liquid chromatographic method in the determinations. In addition, characterisation of ripening stages of four hybrids was aimed.

## 2. Material and Methods

### 2.1. Plant Material

The plants were cultivated with convention horticultural practices in the experimental field of Szent István University, Gödöllő, Hungary. Bandai F_1_ (Bandai) and Beibeihong 695 F_1_ (Beibeihong) which belong to* Capsicum frutescens* and Lolo 736 F_1_ (Lolo) and Chili 3735 F_1_ (C3735) which belong to* Capsicum annuum* were all purchased from East-West Seeds Company, from Thailand, while Star Flame and Fire Flame (both* Capsicum annuum*) were purchased from Seminis, Hungary. The pods of Bandai, Beibeihong, Lolo, C3735, and Fire Flame are red when fully ripe, while Star Flame has vivid yellow pods. Peppers with intermediate pungency level were selected for the investigation because those have multiple utilization methods. Those peppers involved in the recent study have limited data available for breeders and growers; thus, it makes them important for research work. Star Flame and Fire Flame are commercially available in certain European countries but not yet in Hungary.

### 2.2. Capsaicinoid Determination

The determination of capsaicinoid content was made following the method of Daood et al. [27]. Three grams of well-blended pepper sample were crushed in a crucible mortar with quartz sand. To the macerate 50 mL of methanol (analytical grade) was added and the mixture was then transferred to a 100 mL Erlenmeyer flask. The mixture was subjected to 4 min long ultrasonication (Raypa, Turkey) and then filtered through filter paper (Munktell, Germany). The filtrate was more purified by passing through a 0.45 mm PTFE syringe filter before injection on the HPLC column.

After suitable dilution, the extract was injected to Nucleodur C18, Cross-Linked (ISIS, from Macherey-Nagel, Düren, Germany). The separation was performed with isocratic elution of 50 : 50 water-acetonitrile and a flow rate of 0.8 mL/min. Fluorometric detection of capsaicinoid was carried out at EX: 280 nm and EM: 320 nm.

Peaks referring to different capsaicinoids were identified by comparing retention times and mass data (Daood et al. [27]) of standard material (purified from pungent red pepper, with 99% purity, by Plantakem Ltd., Sándorfalva, Hungary) with those appearing on chromatogram of samples. Capsaicinoid compounds are referred as follows: nordihydrocapsaicin (NDC), capsaicin (CAP), dihydrocapsaicin (DC), homocapsaicin 1-2 (HCAP1-2), and homodihydrocapsaicin 1-2 (HDC1-2). Scoville heat unit (SHU) was calculated by the following algorithm:

(1) CAP×16,1+DC×16,1+NDC×9,3+HCAP1+HCAP2×8,6=Scoville  heat  unit.

All variables are expressed in *μ*g/g dry weight basis [28].

### 2.3. Polyphenol Determination

Five grams of well-blended pepper sample were replaced into an Erlenmeyer flask and then 10 mL distilled water was added to the sample and subjected to ultrasonication force using ultrasonic bath for 30 sec. Then, 15 mL of 2% acetic acid in methanol was added to the mixture which was shaken by a mechanical shaker for 15 min. The mixture then was kept overnight at 4°C. Next day after filtrating the mixtures, a further cleanup of the filtrates was made by passing through the mixture a 0.45 *μ*m PTFE HPLC syringe filter. That followed by injection on the HPLC column for the analysis of phenols. Nucleosil C18, 100, Protect-1 (Macherey-Nagel, Düren, Germany), 3 *μ*m, 150 × 4.6 column was used. The gradient elution was done using 1% formic acid (A) in water, acetonitrile (B), and flow rate of 0.6 mL/min. Gradient elution started with 98% A and 2% B and changed in 10 min to 87% A and 13% B and in 5 min to 75% A and 25% B and then in 15 min to 60% A and 40% B; finally it turned in 7 min to 98% A and 2% B. The peaks that appeared on the chromatogram were identified by comparing their retention times and spectral characteristics with available standards such as catechin, quercetin-3-glucoside, kaempferol, luteolin-glucoside, and naringenin-glucoside (Sigma-Aldrich Ltd., Hungary). Quantitation of phenol components having maxima absorption at 280 nm were quantified as catechin equivalent and flavonoids were quantified as kaempferol-equivalent at 350 nm [29, 30]. The standard material was singly injected as external standard as well as being cochromatographed (spiking) with the samples.

### 2.4. Ascorbic Acid Determination

Five grams of well-homogenised sample were disrupted in a crucible mortar with quartz sand. To the macerate 50 mL of metaphosphoric acid (analytical grade) was gradually added and the mixture was then transferred to a 100 mL Erlenmeyer flask closed with stopper and then filtered. The filtrate was purified in addition by passing through a 0.45 mm PTFE syringe filter before injection on HPLC column. The analytical determination of ascorbic acid was performed on C18 Nautilus, 100-5, 150 × 4.6 mm (Macherey-Nagel, Düren, Germany) column with gradient elution of 0.01 M KH2PO4 (A) and acetonitrile (B). The gradient elution started with 1% B in A and changed to 30% B in A in 15 min; then; it turned to 1% A in B in 5 min. The flow rate was 0.7 mL/min. The highest absorption maxima of ascorbic acid under these conditions were detected at 265 nm. For quantitative determination of ascorbic acid standard materials (Sigma-Aldrich, Budapest, Hungary) were used. Stock solutions and then working solutions were prepared for each compound to make the calibration between concentration and peak area.

### 2.5. HPLC Apparatus

A Hitachi Chromaster HPLC instrument, which consists of a Model 5110 Pump, a Model 5210 Auto Sampler, a Model 5430 Diode Array detector, and a Model 5440 Fluorescence detector, was used for the determination of all compounds.

### 2.6. Validation of Applied Methods

Since the methods used in the different chromatographic determinations are derived from the literature (validated protocols) we dealt with only measuring the limit of detection (LOD) and quantification (LOQ) and linearity curves of different compounds under the conditions of our laboratories. The LOD and LOQ were calculated from standard solutions and samples as the concentrations of analytes at peak/noise of 3 times and 10 times, respectively. Linearity curves were made plotting concentration in *μ*g/mL against peak areas.

### 2.7. Dry Matter Determination

Three grams of fresh pepper samples were dried at 65°C until constant weight. The dry matter content was measured as a proportion of fresh and dried fruit weight.

### 2.8. Statistical Analyses

For each independent variable a one-way linear model (LM) was fitted, where “ripening stage” was set as explanatory (factor) variable. Prior to model fitting assumptions were checked by plot diagnosis. In the analysis of the major compounds (SHU, CAP, naringenin-diglucoside, ascorbic acid, and dry matter) among the six hybrids another LM was made, where “hybrid” was set as explanatory (factor) variable. Post hoc comparison was made by Tukey HSD test. All statistical analyses were performed in IBM SPSS 22 software (IBM Co., USA) and Microsoft Excel (Microsoft Co., USA). *α* was set at 0.05 in the entire study.

## 3. Results and Discussion

To adapt the applied chromatographic protocols under the conditions of our laboratories, certain parameters such as LOD, LOQ, and linearity curve were studied. The values depicted in Table 1 show that the used methods are accurate enough to carry on precise and sensitive determination of polyphenols, capsaicinoids, and ascorbic acid. This statement is based on the low levels of LOQ, LOD found for all tested compounds. The concentration of such compounds in our samples is much higher than the levels of LOQ and LOD. Moreover, values obtained for regression coefficient indicated that the methods can be applied at wide range of concentrations for different compounds in chili samples.

### 3.1. Pungency

**Figure 1:**
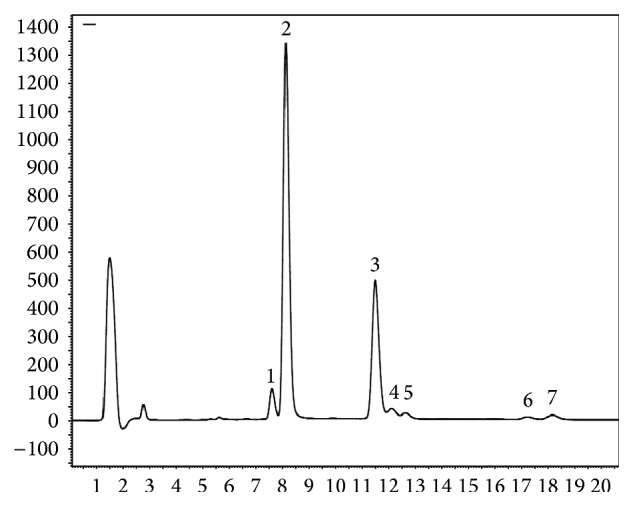
HPLC profile of capsaicinoid components separated from red stage of Bandai hybrid using cross-linked C18 column with acetonitrile-water elution and fluorescence detection. 1: NDC, 2: CAP, 3: DC, 4: HCAP1, 5: HCAP2, 6: HDC1, and 7: HDC2. For more information see text.

The major components evolving pungency in our hybrids are NDC, CAP, and DC. Besides, we could identify the homologues of CAP and DC which are HCAP1, HCAP2 and HDC1, HDC2, respectively (Figure 1). All of them are branched-chain alkyl vanillylamides. Kozukue et al. [31] detected the 7 compounds, in addition to nonivamide which is a straight-chain nonoyl vanillylamide analog of CAP [1]. In Beibeihong advance in ripening did not affect the major capsaicinoids (CAP, NDC, and DC shown in Table 2), while it influenced HCAP1 and HDC1 (both *p* ≤ 0.032) including a slight decrease from green to colour-break stage and then a low increase at the final stage. In Bandai, unlike Beibeihong the ripening affected the major and minor capsaicinoids as well (all *p* ≤ 0.027). The changing of CAP included a notable decrease between the initial stage and the colour-break stage. On DC, NDC, and HDC2 a gradual decrease was measured. A straight increasing of HDC1 was observed, while on HPC1 the same tendency like that in HPC1 of Beibeihong was observed.

Focusing on the major compounds of capsaicinoids, Bandai hybrid could be characterised with pungency loss, while in Beibeihong those compounds did not change during ripening. In the study by Gnayfeed et al. [12] CAP reached the highest value in F-03 cultivar (*C. annuum*) at the initial green stage, similarly found in Bandai, but its content in F-03 did not change significantly with ripening. The obtained results suggest even in the same species (*C. frutescens*) that the hybrids have a different characteristic in ripening regarding capsaicinoid contents. It is in accordance with findings of Merken and Beecher [30] who also measured the maximal capsaicinoid content in 3 different* C. frutescens* peppers in 3 variant times after flower budding.

In Lolo the ripening slightly affected but not significantly CAP, while it increased HCAP2 (*p* = 0.001). After the colour-break stage the amount of HCAP1 decreased (*p* = 0.008) to undetectable level. In C3735 ripening decreased NDC, CAP, DC, HDC1 (all *p* ≤ 0.011), and nonmarginally HDC2, while HCAP1 was absent or under detection limit at all ripening stages. Therefore, most of the compounds showed a decreasing tendency during ripening of C3735, so a remarkable pungency loss was observed. On the contrary, those compounds remained unchanged in Lolo.

Iwai found the peak 40 days after flowering and then a gradual decrease of capsaicinoid content in a* C. annuum* pepper. Because of the different scale used by Iwai et al. [32], it is difficult to compare to our data, but probably the 40 days after flowering is roughly equal to the green stage we used. Gnayfeed et al. [12] observed in* C. annuum* cultivars that capsaicinoids reached maximum level at the colour-break stage and then started declining in Hungarian spice pepper (*C. annuum*), which is a characteristic of pungency change that we did not observe. The change in capsaicin content during ripening of pepper may relate to activity of some enzymes that interfere in the ripening dynamics. The amount of capsaicinoids has been investigated in relation with several enzymes [10, 33, 34]. Contreras-Padilla and Yahia [10] showed that peroxidase activity started increasing, when the amount of capsaicinoid started to decrease in Habanero and de Arbol, while in Piquin it began to increase before the decrease of capsaicinoid. They concluded that peroxidase enzyme is involved in capsaicinoid degradation and that attribution is a genotypic characteristic. Iwai et al. [32] found higher phenylalanine ammonia-lyase activity in green stage than in red stage. In addition, Bernal et al. [33] observed that the operation of capsaicinoid synthetase enzyme is more influenced by the availability of precursors and the conditions of forming than its substrate specificity. The capsaicinoid composition and content are the result of the above referred enzymes.

A study concerning the maturation of Habanero (*C. chinense*) proved that green pod contains four times less capsaicin than ripe red ones [13], while we found less difference and even more capsaicin in green stage (e.g., Bandai); however, none of our investigated hybrids belong to* C. chinense*. They also reported that DC content is seven times less in green pods as compared to red ones, while we found only a slight decrease of DC between the green and red stages.

### 3.2. Polyphenols

Since there is no available standard for myricetin and vanillic acid in our laboratory, they were tentatively identified based on comparison of their spectral characteristics and retention behaviour on the HPLC column with those found in the literature.

**Figure 2:**
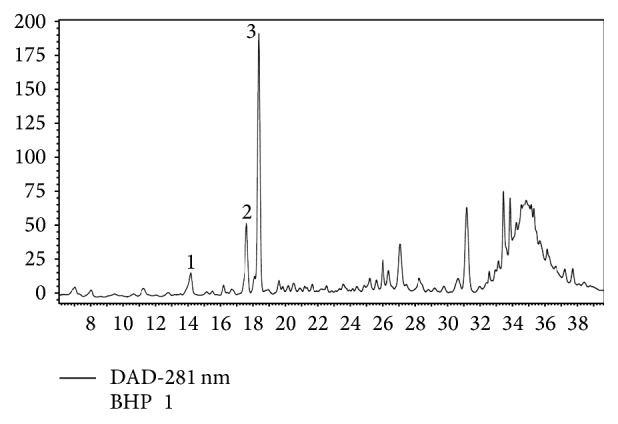
HPLC profile of polyphenols detected separated on Protect-1 C18 column and detected at 280 nm. 1: vanillic acid-derivative, 2: catechin, and 3: naringenin-diglucoside.

**Figure 3:**
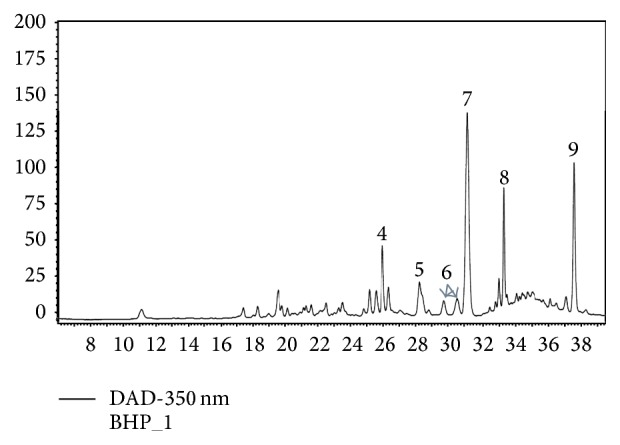
HPLC profile of polyphenols detected separated on Protect-1 C18 column and detected at 350 nm. 4: luteolin-rutinoside, 5: quercetin-glucoside, 6: quercetin-glycosides (the sum of these compounds is used in Table 3), 7: luteolin-glucoside, 8: myricetin, and 9: kaempferol-derivative.

Due to the high content of vanillic acid-derivative, catechin, and naringenin-diglucoside, those compounds were found to be the dominant polyphenols, which have maxima absorption at 280 nm (Figure 2). The minor compounds were luteolin-rutinoside, quercetin-glucoside, quercetin-glycosides, myricetin, and kaempferol-derivative; all were detected with maxima absorption at 350 nm and also luteolin-glucoside occurs in higher concentration and is detected at 350 nm (Figure 3).

In Beibeihong, ripening increased catechin, luteolin-rutinoside, quercetin compounds, myricetin, and kaempferol-derivative (all *p* ≤ 0.02 shown in Table 3), while it decreased vanillic acid content (*p* < 0.001). In Bandai ripening increased all compounds (all *p* ≤ 0.002) except vanillic acid and luteolin-rutinoside which statistically remained unchanged during ripening stages. In quercetin-glucoside, myricetin, and kaempferol-derivative the highest values were measured in the middle of the ripening. Most of the studies regarding polyphenol constitution of pungent pepper focus on the green (initial) and red (final) ripe stages but omit the intermediate or colour-break stage. Howard et al. [20] found that quercetin decreased, while luteolin did not change with ripening of Tabasco (*C. frutescens*). On the contrary, we found an increase of quercetin-related compounds in both* C. frutescens* hybrids and also an increase of luteolin-rutinoside in Beibeihong and of luteolin-glucoside in Bandai.

In Lolo the ripening significantly decreased vanillic acid (*p* < 0.001) but increased catechin, luteolin-rutinoside, luteolin-glucoside, and myricetin (all *p* < 0.001). In C3735 vanillic acid decreased (*p* = 0.007) while catechin, naringenin-diglucoside, and myricetin increased (all *p* ≤ 0.019). Howard et al. stated that quercetin had either increasing or decreasing tendency depending on cultivar; also no change was observed during maturity stages of certain cultivars on* C. annuum* peppers. We could only confirm the last statement that none of the quercetin-related compounds changed when the pods changed from green to red in* C. annuum* peppers studied.

According to Materska and Perucka [19] the most abundant flavonoid compounds in the green stage were quertecin-3-O-L-rhamnoside and luteolin-related compounds, and with ripening those phytochemicals decreased. In the present work particularly in red stage contained higher amounts of luteolin-related in Lolo, while in quercetin-glycoside content no change was detected in both* C. annuum* hybrids.

The disappearances of flavonoids are parallel to capsaicinoids accumulation [35] because the synthesis of flavonoids may converge with the capsaicinoid pathways [36]. The only nonflavonoid phenolic acid detected in our peppers is vanillic acid, and it is the only polyphenol compound which decreased or stayed unchanged during ripening, while the flavonoids mostly increased with advance of ripening. At the same time the major capsaicinoids generally decreased or did not change even with ripening. Kawada and Iwai [37] found a direct relation between DC and vanillic acid; they fed rats with DC and then detected vanillic acid in a notable amount in the urine of the rats. This experiment may also support our findings that vanillic acid is certainly related to capsaicinoids and has similar dynamics during ripening in pungent pepper.

According to Tsao [14], flavonols (kaempferol, quercetin, and myricetin) consist of highly conjugated bindings and a 3-hydroxy group, whose attributions are considered very important in evolving high antioxidant activity. In our hybrids the highest levels of the latter flavonoids were obtained at the orange or red stage that makes the pepper of higher nutritive value.

### 3.3. Ascorbic Acid

**Figure 4:**
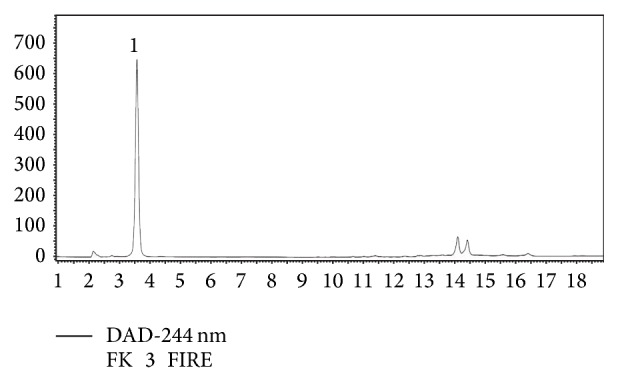
HPLC profile of vitamin C determination. The separation was performed on C18 Nautilus column with PDA detection at 244 nm. 1: L-ascorbic acid.

By the applied HPLC method only L-ascorbic acid was found in the extract of all hybrids (Figure 4). It was found that ascorbic acid increased during ripening in all hybrids (*p* ≤ 0.001 shown in Table 4). In Beibeihong and Bandai after green stage a more notable increase was observed than after the colour-break stage where the ascorbic acid gradually increased, while in Bandai at the red stage the average of ascorbic acid was less than in orange stage. In Lolo the green and colour-break stage did not differ significantly, while the red stage contained the most. In C3735 a straight increase was observed. The increasing tendency in the investigated hybrids is in accordance with that found in previous works [12, 20, 24, 25] which concluded that the more ripened the pods were, the more ascorbic acid could be measured from them. With ripening the pepper pods store more reducing sugars [36], which are the precursors of L-ascorbic acid [38], and that explains the increasing vitamin C content with ripening in all hybrids included in our study. On the contrary, Shaha et al. [18] showed a different dynamics of the ascorbic acid accumulation, because they found the highest level in yellow (intermediate) stage and the declining level in the red mature stage. That agrees with our finding in Bandai, where the highest average values (1005.2 ± 100.73 *μ*g/g) were observed in the orange or colour-break stage (937.9 ± 78.04 *μ*g/g), although these are not significantly higher than that determined in red stage (787.4 ± 131.21 *μ*g/g). Probably it is also due to the high standard deviation present in the red stage.

The recommended daily allowance (RDA) is 60 *μ*g FW; according to Dias [39] 100 g fresh chili provides about 143.7 *μ*g vitamin C. Focusing on the hybrids of the recent study at the green stage all of them failed to reach this value, while at colour-break stage Beibeihong and C3735 reached it and finally at the red stage all of them achieved the RDA.

### 3.4. Comparison of Major Compounds among the 6 Hybrids

The comparison among the hybrids has been done on the main parameters: CAP, ascorbic acid, naringenin-diglucoside, Scoville heat unit, and dry matter (shown in Table 5), at the final stage of the hybrids, which is generally considered as the most valuable in nutrition and having the most processing possibility. A higher dry matter signifies a better fruit quality and also a higher nutritional concentration when fresh weight basis is used to express nutritional parameters. We measured 25–30% dry matter content in* C. frutescens*, which produces more seeds and smaller pods, while in the peppers belonging to* C. annuum* this value lessens to 14.1–15.8%.

The CAP content was found to be statistically the same in all red coloured* C. annuum* hybrids, while the yellow hybrid Star Flame (234.3 ± 45.23 *μ*g/g) contained more, and Bandai (1176.1 ± 112.1 *μ*g/g) the most (*p* < 0.001). Our findings roughly agree with the result of Sanatombi and Sharma [40] who showed that the cultivars belonging to* C. annuum* contain less capsaicin than others of* Capsicum frutescens*. Beibeihong was an exception, because it statistically contained the same amount as* C. annuum* hybrids. Focusing on the Scoville heat units, the highest CAP value in Bandai corresponds to the highest SHU (98090.8 ± 9920.74) observed among the hybrids investigated. Bernal et al. [33] measured 87300–276500 SHU in ripe* C. frutescens* peppers, but in Bandai hybrid the value found was close to the lower level determined by the authors. Among* C. annuum* hybrids, Star Flame was found to be a prominent pepper regarding SHU (66201.2 ± 7132.51) comparing to the measurements of Topuz and Ozdemir [41] 9720 ± 2061.8 and Giuffrida et al. [42] 21034 ± 3579. Beibeihong and Bandai have not been investigated by pungency profiles before. Comparing to Tabasco (also belonging to* C. frutescens*) the SHU measured by Giuffrida et al. [42] (21348 ± 867) is below our values of the latter hybrids, although CAP content determined by Giuffrida et al. [42] (917 ± 34 *μ*g/g) is between the values measured in Bandai (1176.1 ± 112.1 *μ*g/g) and Beibeihong (311.8 ± 63.25 *μ*g/g). Interestingly, Bandai hybrid had the highest CAP content at the same time; it also had the lowest ascorbic acid amount. Topuz and Ozdemir [41] described in pungent peppers that the content of ascorbic acid and capsaicinoid is positively related, which we could not underline in case of Bandai. The highest ascorbic acid was measured in ripe Fire Flame (3689.4 ± 160.61 *μ*g/g) and this value is well above the one measured in Hungarian spice pepper where approximately 1800 *μ*g/g converted to fresh weight basis [12], and it is more than the one detected in New Mexican-type chili peppers 2766 *μ*g/g [25].

Naringenin-diglucoside content ranged from 93.5 ± 4.26 to 368.8 ± 30.77 *μ*g/g and had higher values in* C. frutescens* hybrids compared to* C. annuum* hybrids, probably because of the higher dry matter content of such peppers. Naringenin (belonging to flavanones), being an initial compound in the chain of flavonoid synthesis [14], explains the high content present in our samples. Other studies found also naringenin-glucosides as a dominant flavonoid in peel of pungent pepper [43] and in sweet pepper alike [44].

## 4. Conclusion

The investigated new hybrids can be regarded to be good sources of phytochemicals for future applications. We recommend using the red coloured hybrid Fire Flame to produce chili products with high content of vitamin C. On the other hand, when heat principles (capsaicinoid) for food and pharmaceutical industries are required, the use of Star Flame and Bandai can be suggested, as they contain a level of capsaicin around 440.8 ± 17.22 *μ*g/g and 1610.2 ± 91.46 *μ*g/g, respectively. In order to get the maximum level of the bioactive phytochemicals such as vitamin C, capsaicinoid, and polyphenol it is important to characterize the ripening dynamics of each of these new hybrids. For example, the highest level of capsaicin could be found in the green stage of ripening of Bandai and C3735 hybrids, while in the other hybrids pungency was similar in all ripening stages.

## Figures and Tables

**Table 1:** Some validation parameters for the HPLC determinations of the major polyphenols, ascorbic acid, and capsaicinoids.

	LOD *μ*g/mL	LOQ *μ*g/mL	Linearity range *μ*g/mL	Linearity curve	*R* ^2^
Catechin	2.625	8.75	0–50	*y* = 0.331*x* − 2.5895	0.899
Naringenin-diglucoside	0.0318	0.106	0–50	*y* = 0.3906*x* − 3.0556	0.899
Quercetin-3-glucoside	1.083	3.61	0–50	*y* = 0.2188*x* − 0.781	0.983
Luteolin-glucoside	1.018	3.39	0–50	*y* = 0.1912*x* − 0.381	0.979
Kaempferol-derivative	0.0208	0.069	0–50	*y* = 0.4402*x* − 3.444	0.899
Ascorbic acid	2.500	0.750	30–120	*y* = 0.2736*x* − 2.4305	0.997
Nordihydrocapsaicin^*∗*^	0.003^*∗*^	0.008^*∗*^	0–0.07–1.1^*∗*^	*y* = 2000 + 07*x* + 3000 + 06^*∗*^	0.997^*∗*^
Capsaicin^*∗*^	0.004^*∗*^	0.01^*∗*^	0.1–5^*∗*^	*y* = 2000 + 07*x* + 3000 + 06^*∗*^	0.998^*∗*^
Dihydrocapsaicin^*∗*^	0.002^*∗*^	0.007^*∗*^	0.3–6^*∗*^	*y* = 2000 + 07*x* + 3000 + 06^*∗*^	0.998^*∗*^

^*∗*^From previously published research work on HPLC determination of capsaicinoids by Daood et al. [27].

**Table 2:** Change in content of capsaicinoid compounds in chili hybrids as a function of ripening. The values represent means in *μ*g/g fresh base weight ± standard deviation (*n* = 3).

Hybrid	Ripening stage	NDC (*μ*g/g)	CAP (*μ*g/g)	DC (*μ*g/g)	HCAP1 (*μ*g/g)	HCAP2 (*μ*g/g)	HDC1 (*μ*g/g)	HDC2 (*μ*g/g)
Beibeihong	Green	51.8 ± 3.90a	294.5 ± 19.72a	326.5 ± 51.20a	3.5 ± 2.01ab	20.0 ± 2.37a	10.6 ± 3.01ab	26.3 ± 2.60a
	Colour-breaker	60.6 ± 15.01a	254.6 ± 31.90a	263.4 ± 25.92a	1.8 ± 0.70a	25.3 ± 5.13a	9.0 ± 0.82a	29.5 ± 6.42a
	Orange	61.7 ± 6.65a	261.9 ± 26.12a	269.3 ± 14.06a	3.6 ± 0.97ab	28.5 ± 4.64a	10.7 ± 0.50ab	26.7 ± 2.09a
	Red	63.2 ± 15.12a	311.8 ± 63.25a	272.7 ± 74.99a	5.7 ± 0.51b	23.7 ± 3.62a	13.9 ± 0.5b	23.9 ± 3.79a
	*F*-value	0.61	1.44	1.12	5.41	2.26	4.89	0.94
	*p* value	0.626	0.302	0.394	0.025	0.158	0.032	0.464

Bandai	Green	102.9 ± 14.17ab	1610.2 ± 91.46b	780 ± 36.03b	13.1 ± 5.61ab	8.9 ± 0.99a	12.8 ± 1.22a	30.2 ± 2.29ab
	Colour-breaker	102.2 ± 1.21ab	1182.2 ± 82.56a	725.2 ± 32.03ab	6.3 ± 1.83a	18.5 ± 3.69b	12.7 ± 0.57a	31 ± 3.41ab
	Orange	115.6 ± 5.26b	1104.9 ± 77.27a	635.2 ± 32.36a	11.5 ± 0.51ab	27.2 ± 3.33c	15 ± 0.46ab	37.9 ± 3.41b
	Red	81.5 ± 6.91a	1176.1 ± 112.1a	600.4 ± 87.11a	20.1 ± 6.24b	14.3 ± 2.39ab	16.3 ± 2.06b	27.3 ± 3.34a
	*F*-value	8.59	18.93	7.40	5.27	22.70	5.95	6.10
	*p* value	0.007	0.001	0.011	0.027	<0.001	0.020	0.018

Lolo	Green	18.7 ± 2.42a	222.5 ± 69.33a	139.2 ± 50.97a	0.5 ± 0.23b	0.4 ± 0.08a	1.8 ± 0.15a	9.2 ± 1.19a
	Colour-breaker	26.2 ± 3.94a	95.5 ± 4.15a	96.7 ± 9.29a	0.4 ± 0.04b	2.4 ± 0.77b	1.9 ± 0.08a	12.6 ± 1.31a
	Red	22.2 ± 5.14a	197 ± 92.13a	119.8 ± 53.03a	0 ± 0a	3.2 ± 0.15b	1.9 ± 0.27a	12.5 ± 3.27a
	*F*-value	2.69	3.05	0.74	12.12	29.94	0.86	2.54
	*p* value	0.146	0.122	0.516	0.008	0.001	0.467	0.159

C3735	Green	31.3 ± 1.46b	259.6 ± 39.15b	183.4 ± 23.27b	UDL	7 ± 0.79a	1.6 ± 0.21b	8.2 ± 0.58ab
	Colour-breaker	35.9 ± 1.64b	168.9 ± 33.86ab	148.2 ± 24.21b	UDL	12.6 ± 4.16a	1.9 ± 0.08b	9.8 ± 1.01b
	Red	18.2 ± 6.21a	126.3 ± 35.95a	88.9 ± 6.38a	UDL	12.6 ± 3.7a	1.3 ± 0.07a	7.2 ± 1.31a
	*F*-value	17.39	10.50	17.58	—	3.00	15.03	5.00
	*p* value	0.003	0.011	0.003	—	0.125	0.005	0.053

Fire Flame	Red	15.5 ± 3.28	234.3 ± 45.23	109.7 ± 19.9	1.2 ± 0.13	1 ± 0.23	0.9 ± 0.13	5.9 ± 1.24

Star Flame	Yellow	21.9 ± 5.36	440.8 ± 17.22	135.9 ± 20.28	2.5 ± 0.08	0.2 ± 0.08	1.4 ± 0.25	6.4 ± 1.10

The same letter indicates no significant difference in capsaicinoid content between ripening stages in the given hybrid according to Tukey HSD post hoc test; UDL: under detection limit.

**Table 3:** Change in content of polyphenol compounds in different chili hybrids as a function of ripening. The values represent means in *μ*g/g fresh weight base ± standard deviation (*n* = 3).

Hybrid	Ripening stage	Vanillic acid-derivative (*μ*g/g)	Catechin (*μ*g/g)	Naringenin-diglucoside (*μ*g/g)	Luteolin-rutinoside (*μ*g/g)	Quercetin-glucoside (*μ*g/g)	Quercetin-glycosides (*μ*g/g)	Luteolin-glucoside (*μ*g/g)	Myricetin (*μ*g/g)	Kaempferol-derivative (*μ*g/g)
Beibeihong	Green	109.5 ± 9.84b	50.4 ± 1.86a	349.5 ± 13.09a	11.3 ± 0.53a	12.5 ± 2.07a	3.7 ± 0.14a	62.9 ± 2.78a	10.3 ± 0.74a	22.6 ± 1.13a
	Colour-breaker	145.7 ± 9.71c	135.3 ± 3.97b	477.4 ± 52.69b	8.5 ± 0.73a	13.4 ± 1.19a	4.7 ± 0.12ab	79 ± 2.78a	23.5 ± 1.13b	67.3 ± 5.26c
	Orange	114.9 ± 9.66b	153.5 ± 5.56c	431.3 ± 39.72ab	9.7 ± 0.67a	9.1 ± 0.07a	4.9 ± 0.62b	84.6 ± 13.18a	24.9 ± 0.46b	69.7 ± 3.86c
	Red	79.2 ± 11.08a	132.7 ± 3.85b	368.8 ± 30.77a	17.0 ± 3.37b	21.1 ± 2.69b	5.5 ± 0.68b	90.1 ± 16.97a	31.9 ± 6.77b	51.1 ± 5.22b
	*F*-value	21.88	390.61	7.54	13.47	23.64	7.62	3.45	20.45	79.18
	*p* value	<0.001	<0.001	0.01	0.02	<0.001	0.01	0.071	<0.001	<0.001

Bandai	Green	96.3 ± 0.25a	96.9 ± 7.67a	130.3 ± 4.82a	13.2 ± 1.06a	4.6 ± 0.66a	5.6 ± 0.24a	84.6 ± 2.37a	25.7 ± 1.58a	62.8 ± 3.33a
	Colour-breaker	89.7 ± 7.98a	134.6 ± 17.02b	202 ± 17.77b	15.1 ± 3.96a	14.2 ± 0.61ab	7.8 ± 0.91ab	91.4 ± 13.75a	117.1 ± 8.21b	289.5 ± 45.59bc
	Orange	92 ± 14.56a	166.4 ± 17.16b	254.2 ± 38.95b	14.8 ± 3.68a	17.4 ± 2.99c	10.2 ± 1.38bc	107.3 ± 23.49a	110.4 ± 19.41b	307.8 ± 53.01c
	Red	101.6 ± 5.67a	175.2 ± 12.32c	276.5 ± 16.65c	21.6 ± 10.69a	12.8 ± 0.97b	11.9 ± 1.15c	157.8 ± 15.26b	52.9 ± 12.94a	200.7 ± 20.83b
	*F*-value	1.05	19.03	23.73	1.15	33.24	22.39	13.41	38.70	28.15
	*p* value	0.42	<0.001	<0.001	0.385	<0.001	<0.001	0.002	<0.001	<0.001

Lolo	Green	72.2 ± 0.85c	45 ± 2.71a	116.8 ± 7.28a	2.1 ± 0.49a	5.7 ± 0.83b	5 ± 0.78b	25.7 ± 5.05a	2 ± 0.51a	UDL
	Colour-breaker	50.3 ± 2.85a	51.4 ± 3.09a	160.8 ± 14.93b	3.2 ± 0.36a	2.8 ± 0.08a	2.9 ± 1.03a	53.4 ± 4.52b	4.8 ± 0.62b	UDL
	Red	64.2 ± 4.15b	171.5 ± 6.14b	117.8 ± 7.18a	7.3 ± 1.22b	6.1 ± 1.28b	4.5 ± 0.28ab	75.9 ± 2.48c	8.9 ± 0.57c	9.1 ± 3.77
	*F*-value	42.59	836.79	17.35	37.55	12.72	6.49	108.95	113.35	—
	*p* value	<0.001	<0.001	0.003	<0.001	0.007	0.032	<0.001	<0.001	—

C3753	Green	73.1 ± 5.46b	22.6 ± 1.28a	123.6 ± 6.23a	2 ± 0.7a	1.7 ± 0.39a	16.8 ± 2.38a	17 ± 2.57a	3.8 ± 0.96a	UDL
	Colour-breaker	51 ± 8.55a	64.2 ± 10.49b	148.3 ± 40.01ab	1.5 ± 0.37a	1.3 ± 0.24a	17.3 ± 3.44a	13.5 ± 2.66a	10 ± 1.09b	21 ± 1.71a
	Red	56 ± 6.67a	124.5 ± 6.18c	217.2 ± 8.36b	UDL	1.1 ± 0.23a	19.8 ± 2.55a	17.5 ± 2.78a	11.3 ± 0.94b	16.4 ± 2.85a
	*F*-value	13.06	157.47	8.32	0.44	2.84	0.95	1.99	48.91	6.007
	*p* value	0.007	<0.001	0.019	0.661	0.135	0.44	0.12	<0.001	0.070

Fire Flame	Red	27.8 ± 2.07	26.6 ± 1.40	141.6 ± 4.17	2.8 ± 0.27	2.7 ± 0.16	4.2 ± 0.22	44.4 ± 2.76	18.2 ± 0.43	UDL

Star Flame	Yellow	24.2 ± 1.37	17.6 ± 0.24	93.5 ± 4.33	9.5 ± 0.47	2.0 ± 0.14	4.1 ± 0.62	60.6 ± 1.34	18.4 ± 1.29	UDL

The same letter indicates no significant difference in polyphenol content between ripening stages in the given hybrid according to Tukey HSD post hoc test; UDL: under detection limit.

**Table 4:** Change in content of ascorbic acid in different chili hybrids as a function of ripening. The values represent means in *μ*g/g fresh weight base ± standard deviation (*n* = 3).

Hybrid	Ripening stage	Ascorbic acid (*μ*g/g)
Beibeihong	Green	355 ± 64.85a
	Colour-breaker	1503.4 ± 358.31b
	Orange	2085.7 ± 252.2bc
	Red	2483.8 ± 570.74c
	*F*-value	19.74
	*p* value	<0.001

Bandai	Green	329.5 ± 58.88a
	Colour-breaker	937.9 ± 78.04b
	Orange	1005.2 ± 100.73b
	Red	787.4 ± 131.21b
	*F*-value	30.09
	*p* value	<0.001

Lolo	Green	111.3 ± 14.01a
	Colour-breaker	451.5 ± 115.56a
	Red	1940.9 ± 533.57b
	*F*-value	28.57
	*p* value	0.001

C3735	Green	315.1 ± 59.91a
	Colour-breaker	1522.5 ± 127.47b
	Red	2468.2 ± 58.93c
	*F*-value	449.65
	*p* value	<0.001

Fire Flame	Red	3689.4 ± 39.50

Star Flame	Yellow	3154.8 ± 160.61

The same letter indicates no significant difference in ascorbic acid content between ripening stages in the given hybrid according to Tukey HSD post hoc test.

**Table 5:** Capsaicin, ascorbic acid, and naringenin-diglucoside content (*μ*g/g fresh weight base), pungency unit of Scoville, and dry matter of different chili hybrids. The values represent means ± standard deviation (*n* = 3).

Hybrid	Capsaicin (*μ*g/g)	Scoville heat unit	Ascorbic acid (*μ*g/g)	Naringenin-diglucoside (*μ*g/g)	Dry matter
Beibeihong	311.8 ± 63.25ab	37999.8 ± 5761.66a	2483.8 ± 570.74bc	368.8 ± 30.77f	25.8 ± 0.82d
Bandai	1176.1 ± 112.1c	98090.8 ± 9920.74c	787.4 ± 131.21a	276.5 ± 16.65e	30.2 ± 0.41c
Lolo	197 ± 92.13a	33188.2 ± 5229.83a	1940.9 ± 533.57b	117.8 ± 7.18ab	14.0 ± 0.61a
C3735	126.3 ± 35.95a	23730.9 ± 3174.95a	2468.2 ± 58.93bc	217.2 ± 8.36d	15.8 ± 0.93b
Fire Flame	234.3 ± 45.23a	40417.3 ± 7830.33a	3689.4 ± 160.61d	141.6 ± 4.19c	14.1 ± 0.34ab
Star Flame	440.8 ± 17.22b	66201.2 ± 7132.51b	3154.8 ± 160.61cd	93.5 ± 4.26a	14.4 ± 0.58ab
*F*-value	94.64	48.43	27.62	146.17	357.48
*p* value	<0.001	<0.001	<0.001	<0.001	<0.001

The same letter indicates no significant difference in the major components between the fully ripe stages of the 6 hybrids according to Tukey HSD post hoc test.

## References

[1] Mózsik G., Dömötör A., Past T. (2009). Chemical taxonomy of the functional parts of the *Capsicums*. *Capsaicinoids*.

[2] DeWitt D., Bosland P. W. (2009). Capsaicin and the quest for the world's hottest pepper. *The Complete Chile Pepper Book*.

[3] Mózsik G., Szolcsányi J., Rácz I. (2005). Gastroprotection induced by capsaicin in healthy human subjects. *World Journal of Gastroenterology*.

[4] Wood J. N., Winter J., James I. F., Rang H., Yeats J., Bevan S. (1988). Capsaicin-induced ion fluxes in dorsal root ganglion cells in culture. *The Journal of Neuroscience*.

[5] Wachtel R. E. (1999). Capsaicin. *Regional Anesthesia and Pain Medicine*.

[6] Medina-Lara F., Echevarría-Machado I., Pacheco-Arjona R., Ruiz-Lau N., Guzmán-Antonio A., Martinez-Estevez M. (2008). Influence of nitrogen and potassium fertilization on fruiting and capsaicin content in Habanero pepper (*Capsicum chinense* Jacq.). *HortScience*.

[7] Sung Y., Chang Y. Y., Ting N. L. (2005). Capsaicin biosynthesis in water-stressed hot pepper fruits. *Botanical Bulletin of Academia Sinica*.

[8] Gurung T., Techawongstien S., Suriharn B., Techawongstien S. (2012). Stability analysis of yield and capsaicinoids content in chili (*Capsicum* spp.) grown across six environments. *Euphytica*.

[9] Harvell K. P., Bosland P. W. (1997). The environment produces a significant effect on pungency of chiles. *Hortscience*.

[10] Contreras-Padilla M., Yahia E. M. (1998). Changes in capsaicinoids during development, maturation, and senescence of chile peppers and relation with peroxidase activity. *Journal of Agricultural and Food Chemistry*.

[11] Shan-Han C., Shen-Kui H. E., Wen-Bin C., Yan-Ge W. U. (2009). Detection of capsaicin and dihydrocapsaicin content and analysis of pungency degree in different pepper genotypes. *Natural Science Journal of Hainan University*.

[12] Gnayfeed M. H., Daood H. G., Biacs P. A., Alcaraz C. F. (2001). Content of bioactive compounds in pungent spice red pepper (paprika) as affected by ripening and genotype. *Journal of the Science of Food and Agriculture*.

[13] Menichini F., Tundis R., Bonesi M. (2009). The influence of fruit ripening on the phytochemical content and biological activity of *Capsicum chinense* Jacq. cv Habanero. *Food Chemistry*.

[14] Tsao R. (2010). Chemistry and biochemistry of dietary polyphenols. *Nutrients*.

[15] Iqbal T., Hussain A. I., Chatha S. A., Naqvi S. A., Bokhari T. H. (2013). Antioxidant activity and volatile and phenolic profiles of essential oil and different extracts of wild mint (*Mentha longifoli*a) from the Pakistani Flora. *Journal of Analytical Methods in Chemistry*.

[16] Hernández-Jiménez A., Gil-Muñoz R., Ruiz-García Y., López-Roca J. M., Martinez-Cutillas A., Gómez-Plaza E. (2013). Evaluating the polyphenol profile in three segregating grape (*Vitis vinifera* L.) populations. *Journal of Analytical Methods in Chemistry*.

[17] Harborne J. B., Williams C. A. (2000). Advances in flavonoid research since 1992. *Phytochemistry*.

[18] Shaha R. K., Rahman S., Asrul A. (2013). Bioactive compounds in chilli peppers (*Capsicum annuum* L.) at various ripening (green, yellow and red) stages. *Annals of Biological Research*.

[19] Materska M., Perucka I. (2005). Antioxidant activity of the main phenolic compounds isolated from hot pepper fruit (*Capsicum annuum* L.). *Journal of Agricultural and Food Chemistry*.

[20] Howard L. R., Talcott S. T., Brenes C. H., Villalon B. (2000). Changes in phytochemical and antioxidant activity of selected pepper cultivars (*Capsicum* species) as influenced by maturity. *Journal of Agricultural and Food Chemistry*.

[21] Sauberlich H. E. (1994). Pharmacology of vitamin C. *Annual Review of Nutrition*.

[22] Lutsenko E. A., Carcamo J. M., Golde D. W. (2002). Vitamin C prevents DNA mutation induced by oxidative stress. *The Journal of Biological Chemistry*.

[23] Frei B., Lawson S. (2008). Vitamin C and cancer revisited. *Proceedings of the National Academy of Sciences*.

[24] Bae H., Jayaprakasha G. K., Crosby K. (2014). Ascorbic acid, capsaicinoid, and flavonoid aglycone concentrations as a function of fruit maturity stage in greenhouse-grown peppers. *Journal of Food Composition and Analysis*.

[25] Osuna-García J. A., Wall M. M., Waddell C. A. (1998). Endogenous levels of tocopherols and ascorbic acid during fruit ripening of New Mexican-type chile (*Capsicum annuum* L.) cultivars. *Journal of Agricultural and Food Chemistry*.

[26] Lee S. K., Kader A. A. (2000). Preharvest and postharvest factors influencing vitamin C content of horticultural crops. *Postharvest Biology and Technology*.

[27] Daood H. G., Halász G., Palotás G., Palotás G., Bodai Z., Helyes L. (2014). HPLC determination of capsaicinoids with cross-linked C18 column and buffer-free eluent. *Journal of Chromatographic Science*.

[28] Ziino M., Condurso C., Romeo V., Tripodi G., Verzera A. (2009). Volatile compounds and capsaicinoid content of fresh hot peppers (*Capsicum annuum* L.) of different calabrian varieties. *Journal of the Science of Food and Agriculture*.

[29] Wu S., Dastmalchi K., Long C., Kennelly E. J. (2012). Metabolite profiling of jaboticaba (*Myrciaria cauliflora*) and other dark-colored fruit juices. *Journal of Agricultural and Food Chemistry*.

[30] Merken H. M., Beecher G. R. (2000). Measurement of food flavonoids by high-performance liquid chromatography: a review. *Journal of Agricultural and Food Chemistry*.

[31] Kozukue N., Han J., Kozukue E. (2005). Analysis of eight capsaicinoids in peppers and pepper-containing foods by high-performance liquid chromatography and liquid chromatography-mass spectrometry. *Journal of Agricultural and Food Chemistry*.

[32] Iwai K., Suzuki T., Fujiwake H. (1979). Formation and accumulation of pungent principle of hot pepper fruits, capsaicin and its analogues, in *Capsicum annuun* var. *annuun* cv. karayatsubusa at different growth stages after flowering. *Agricultural and Biological Chemistry*.

[33] Bernal M. A., Calderon A. A., Pedreno M. A., Munoz R., Ros Barcelo A., de Caceres F. M. (1993). Capsaicin oxidation by peroxidase from *Capsicum annuum* (variety *Annuum*) fruits. *Journal of Agricultural and Food Chemistry*.

[34] Gao Y. P., He L. L., Chen J. Q., Gao S., Li X. W., Dong X. K. (2008). Effects of shading on capsaicin and relevant enzymes of fruit in pepper. *Acta Agriculturae Boreali-Sinica*.

[35] Sukrasno N., Yeoman M. (1993). Phenylpropanoid metabolism during growth and development of *Capsicum frutescens* fruits. *Phytochemistry*.

[36] Diaz J., Bernal A., Merino F., Barcelo R. A. (1998). Phenolic metabolism in *Capsicum annuum* L.. *Recent Research Developments in Phytochemistry*.

[37] Kawada T., Iwai K. (1985). *In vivo* and *in vitro* metabolism of dihydrocapsaicin, a pungent principle of hot pepper, in rats. *Agricultural and Biological Chemistry*.

[38] Stone I. (1972). The natural history of ascorbic acid in the evolution of the mammals and primates and its significance for present-day man. *Orthomolecular Psychiatry*.

[39] Dias J. S. (2012). Nutritional quality and health benefits of vegetables: a review. *Food and Nutrition Sciences*.

[40] Sanatombi K., Sharma G. J. (2008). Capsaicin content and pungency of different *Capsicum* spp. cultivars. *Notulae Botanicae Horti Agrobotanici Cluj-Napoca*.

[41] Topuz A., Ozdemir F. (2007). Assessment of carotenoids, capsaicinoids and ascorbic acid composition of some selected pepper cultivars (*Capsicum annuum* L.) grown in Turkey. *Journal of Food Composition and Analysis*.

[42] Giuffrida D., Dugo P., Torre G. (2013). Characterization of 12 *Capsicum* varieties by evaluation of their carotenoid profile and pungency determination. *Food Chemistry*.

[43] Xin X., Fan R., Gong Y., Yuan F., Gao Y. (2014). On-line HPLC-ABTS^•+^ evaluation and HPLC-MS^n^ identification of bioactive compounds in hot pepper peel residues. *European Food Research and Technology*.

[44] Morales-Soto A., Gómez-Caravaca A. M., García-Salas P., Segura-Carretero A., Fernández-Gutiérrez A. (2013). High-performance liquid chromatography coupled to diode array and electrospray time-of-flight mass spectrometry detectors for a comprehensive characterization of phenolic and other polar compounds in three pepper (*Capsicum annuum* L.) samples. *Food Research International*.

